# Letter from Paris

**Published:** 1867-06

**Authors:** J. D'Oyley-Evans


### CORRESPONDENCE.

### ARTICLE VI.

### Letter from Paris.

*Editors American Journal of Dental Science,*

Gentlemen :

A surgeon of some eminence in his profession at Ghent has, recently published an account of a method of treating wounds with dressings of sheet-lead. From the 1st of January, 1864, to the end of May, 1866, Dr. Burggraeve has treated 236 cases in this manner, and only 8 deaths have occurred. His process is exceedingly simple. It consists in washing the wound carefully with luke-warm water, and then covering it with pieces of sheet-lead, which are secured with adhesive plaster. Most of his patients have been workmen injured by machinery, and were too weak to undergo operations owing to the impoverished state of their blood.

"The wound," says Dr. Burggraeve, "whatever may be the amount of contusion, crushing, or laceration, is first washed carefully without detaching or cutting away any portion of flesh, since in the state of torpor it is impossible to say at once which will mortify, and which may be preserved, and one runs the risk either of cutting away too much or too little. It is next surrounded with thin slips of lead, retained in position by sticking plaster. From time to time a jet of warm water is injected under this armor, if we may use the expression, so as to remove the ichor and refresh the parts." In order to watch the progress of the wounds, each sheet of lead may be removed independently of the others. The contact of the metallic lead with the flesh causes no irritation, and the rigidity prevents friction, and excludes the air,—a very important point.

Besides the mechanical action of lead, Dr. Burggraeve thinks that it may also be attended with some physical action, and quotes the well-known effects of Goulard's extract. The author enlarges on the value of this method of treatment in military surgery, where operations must, at least in active service, be somewhat hurried, and many a limb which under ordinary circumstances, might have been preserved, is sacrificed in consequence.

Gun-shot wounds, he says, have much analogy with injuries caused by machinery, and we may reasonably assume that the results will not be dissimilar.

Whatever the theoretical objections to lead bandages may be, they appear at all events to have had a fair trial, and to have been productive of good results.

J. D'OYLEY-EVANS.

---

